# Evolution of Virology: Science History through Milestones and Technological Advancements

**DOI:** 10.3390/v16030374

**Published:** 2024-02-28

**Authors:** Kunlan Zuo, Wanying Gao, Zongzhen Wu, Lei Zhang, Jiafeng Wang, Xuefan Yuan, Chun Li, Qiangyu Xiang, Lu Lu, Huan Liu

**Affiliations:** 1 Department of History of Science and Scientific Archaeology, University of Science and Technology of China, Hefei 230026, China; nemo12@mail.ustc.edu.cn (K.Z.); sa22024037@mail.ustc.edu.cn (W.G.); amoswu@mail.ustc.edu.cn (Z.W.); lorraine@mail.ustc.edu.cn (L.Z.); wjf727@mail.ustc.edu.cn (J.W.); yuanxuefan1110@mail.ustc.edu.cn (X.Y.); wangyilichun@mail.ustc.edu.cn (C.L.); qyxiang@mail.ustc.edu.cn (Q.X.); 2 Key Laboratory of Medical Molecular Virology of MOE/MOH, School of Basic Medical Sciences and Shanghai Public Health Clinical Center, Fudan University, Shanghai 200032, China; lul@fudan.edu.cn; 3 State Key Laboratory of Virology, Wuhan 430072, China

**Keywords:** history of virology, microbiology, biochemistry, genetics, molecular biology

## Abstract

The history of virology, which is marked by transformative breakthroughs, spans microbiology, biochemistry, genetics, and molecular biology. From the development of Jenner’s smallpox vaccine in 1796 to 20th-century innovations such as ultrafiltration and electron microscopy, the field of virology has undergone significant development. In 1898, Beijerinck laid the conceptual foundation for virology, marking a pivotal moment in the evolution of the discipline. Advancements in influenza A virus research in 1933 by Richard Shope furthered our understanding of respiratory pathogens. Additionally, in 1935, Stanley’s determination of viruses as solid particles provided substantial progress in the field of virology. Key milestones include elucidation of reverse transcriptase by Baltimore and Temin in 1970, late 20th-century revelations linking viruses and cancer, and the discovery of HIV by Sinoussi, Montagnier, and Gallo in 1983, which has since shaped AIDS research. In the 21st century, breakthroughs such as gene technology, mRNA vaccines, and phage display tools were achieved in virology, demonstrating its potential for integration with molecular biology. The achievements of COVID-19 vaccines highlight the adaptability of virology to global health.

## 1. Introduction

In 2023, Katalin Kariko and Drew Weissman received the Nobel Prize in Physiology and Medicine for pioneering nucleoside base modification, revolutionizing virology through the development of highly effective mRNA vaccines against the novel coronavirus [1,2]. This innovative mRNA vaccine design, which relies on rapid viral genome sequence analysis, allows rapid vaccine production, enhancing the flexibility of the response to mutations and emerging threats. Beyond their efficacy, mRNA vaccines eliminate live viruses, simplifying production processes and handling.

A virus is a minute infectious particle composed of proteins and nucleic acids. Viruses are incapable of independent existence and require attachment to host cells for replication [3]. Throughout history, viruses have posed threats to human health, giving rise to severe epidemics such as smallpox, plague, and influenza. They also contribute to reduced crop yields and livestock diseases, impacting agriculture. Viruses also play a crucial role in maintaining the biodiversity and ecological balance within ecosystems. Emerging from medicine and bacteriology, virology has a rich history of exploring viral and bacterial diseases. This field delves into the structure, life cycle, transmission, and host interactions of a virus, extending the significance of virology beyond infectious diseases to foundational contributions in molecular biology and genomics. The evolution of virology began in 1898 and can be divided into four periods—microbiology (1934), biochemistry (1935–1954), genetics (1955–1984), and molecular biology (1985-)—according to the main characteristics of the development of virology in the different periods and the technology it relies on. From simple virus observations to intricate molecular research, this evolution parallels the transformative impact of the Industrial Revolution on production methods. Studying the history of virology illuminates the interplay between technology and society, offering insights for epidemic prevention, medical research, and science and technology policy.

Categorized into four periods, the history of virology highlights key breakthroughs, ranging from Martinus Beijerinck’s isolation of the tobacco mosaic virus (TMV) to genetic discoveries and vaccine developments. Virologists have consistently contributed to humanity’s fight against diseases, with ongoing relevance in addressing contemporary global health threats, such as HIV, Ebola, and the novel coronavirus. Kariko and Weissman’s groundbreaking work with mRNA vaccines exemplifies the transformative potential of virology, marking a revolutionary tool against viral threats. This journey through the history of virology underscores its pivotal role in advancing medical knowledge, technology, and global health resilience.

## 2. Microbiology Period (1898–1934)

The microbiological phase of virology development can be traced back to the late 18th century, with Edward Jenner’s pioneering use of viruses for preventive vaccination in 1796. Jenner’s success in smallpox vaccination, employing the vaccinia virus, marked an early milestone in the application of viruses for disease prevention [4]. However, the groundbreaking work conducted by Louis Pasteur and Robert Koch in the 19th century laid the essential foundation for modern virology. In 1885, Pasteur achieved a significant breakthrough by injecting the first rabies vaccine into a rabies-infected boy, contributing significantly to vaccine research and advancing the understanding of viruses [5]. Concurrently, Robert Koch formulated the fundamental principles of pathogenic microbiology, encapsulated in Koch’s Laws, which became instrumental for identifying pathogenic agents in various diseases and propelled the development of virology in the early 20th century [6,7].

The microbiological era faced challenges in studying viruses due to their small size, which limits direct observation under optical microscopes. The advent of ultrafiltration technology marked a revolutionary breakthrough in virus research. Filters such as the Chamberland-Pasteur porcelain filter and the Berkefeld filter facilitated virus screening and isolation, which is crucial for gaining further insights [8]. Researchers ensured the passage of viruses through membranes by adjusting parameters such as the membrane pore size, filtration pressure, temperature, and pH/ion concentration of the suspension fluid and employed prefiltration. Because the virus diameter typically ranges from 20 to 300 nanometers, the dimensions of ultrafiltration membranes typically fall within the range of 1 to 100 nanometers, effectively preventing the passage of most cells and bacteria while allowing the transmission of viruses. This meticulous control facilitated the screening and isolation of viruses through membrane filtration techniques. Key observations in the late 19th century initiated virus research. The observation of tobacco mosaic disease by Adolf Mayer in 1876 suggested that a toxin or tiny bacteria was the cause [9,10]. Dmitry Ivanovsky’s 1892 filtration experiment with a Chamberland filter demonstrated the contagious nature of the disease postfiltration, challenging the prevailing theories [11]. In 1898, Dutch microbiologist Martinus Beijerinck again observed that the infectious agent TMV was present only in cells that were dividing and multiplying, and he thus called TMV the “contagium vivum fluidum” (soluble active microbe), breaking the traditional framework of the germ theory of disease that was widely believed at the time [12]. Beijerinck insisted that viruses were infectious liquids, not microbes, and this theory was later discredited by the American biochemist and virologist Wendell Stanley. Viruses are in fact microparticles. A series of studies on plant and animal diseases that revealed the presence of infectious agents was also the highlight of this period. The isolation of the foot-and-mouth disease virus by Friedrich Loeffler and Paul Frosch in 1898 through filtration contributed significantly to the foundation of animal virology [13]. In 1906, Paul Simond identified the female *Aedes aegypti* mosquito as the vector for yellow fever virus, fundamentally altering the understanding of disease transmission [14].

In the early 20th century, warts were initially perceived as infectious, and in 1907, Giuseppe Ciuffo used the Berkefeld filter to eliminate bacteria, suggesting that the microbial cause was either a virus or extremely tiny bacteria [15]. Research on the viability of viruses gained momentum in 1908, when studies on chicken leukemia by Vilhelm Ellermann and Oluf Bang showed that cell-free filtrates induced disease, and these findings set the groundwork for tumor virology. In 1911, Francis Rous transferred tumors between chickens via a cell-free filtrate, identifying Rous sarcoma virus, the first retrovirus discovered in humans [16]. Bacteriophage exploration commenced in 1917, when Felix d’Herelle discovered bacteriophages, elucidated their life cycle and offered insights for bacteriophage therapy [17]. The history of phage therapy dates back to the early 20th century, when significant advancements in the Soviet Union and France were achieved, and this therapy served as the primary therapeutic approach before the discovery of antibiotics. However, with the advent of antibiotics, this therapy gradually became marginalized in the Western world. In recent years, a resurgence of interest in phage therapy has occurred, driven by the escalating issue of antibiotic resistance. Phage therapy has become a focal point of research and offers an alternative therapeutic approach for addressing contemporary challenges in medicine.

The emergence of tissue culture technology in the early 20th century, which was championed by Alexis Carrel and others, significantly advanced virological research. In 1913–1914, Edna Harde, Clara Israeli, and Robert A. Lambert became the first to grow vaccinia virus in rabbit and guinea pig corneal cells, showing viral neutralization [18]. In the 1920s, Hugh Maitland and other researchers utilized chicken serum and organic salts to cultivate smallpox and other viruses in chicken kidney suspension cultures. By 1931, Albert Woodruff and Ernest Goodpasture successfully used embryonic eggs as viral hosts, providing new tools for studying the life cycles, transmission mechanisms, and modes of infection of viruses [19]. The human influenza A virus was identified in 1933, following the earlier isolation of swine influenza A virus in 1931 by Richard Shope [20]. Subsequent research has significantly advanced virology, immunology, and molecular biology, leading to a deeper understanding of influenza, its pandemics, and the development of preventive measures such as vaccines [21].

In the early 1930s, advancements in colloidal membrane technology allowed accurate estimations of virus particle sizes. William Elford utilized ultrafiltration to determine the particle sizes of Aujeszky’s disease (pseudorabies) virus, human influenza virus, and swine influenza virus. Understanding virus sizes became crucial for vaccine development, infectious disease control, and preventive measures [22].

During the microbiology period, virology advanced from ancient descriptions of infectious diseases to the establishment of its modern concept, guided by ultrafiltration technology. Pioneering research, from Pasteur’s work on rabies to Loeffler and Frosch’s isolation of the foot-and-mouth disease virus, coupled with cultured cells and improvements in ultrafiltration, has illuminated the nature of viruses. Early gaps in the understanding of viruses due to limitations related to filter size and technology were overcome, and the findings revealed that viruses are tiny, granular, and infectious entities. These insights formed the basis for future virology research, guiding vaccine development and disease control.

## 3. Biochemical Period (1935–1954)

Wendell Stanley’s work on tobacco mosaic virus (TMV) in 1935 marked the biochemical period of virology, shifting the focus from the sizes to the molecular mechanisms of viruses. Despite RNA detection errors, Stanley’s isolation of high-purity TMV crystals sparked discussions on life’s nature, laying the groundwork for molecular biology. Awarded the 1946 Nobel Prize, Stanley’s breakthrough, coupled with Bawden and Pirie’s elucidation of the TMV particle structure, unveiled the biological signatures of viruses, which are vital for understanding replication and infectivity [23].

In the late 1920s, revolutionary electron microscopy, which was developed by Ernst Ruska and Max Knoll, surpassed the limitations of light microscopes. The development of electron microscopy began in the late 1920s, when Ernst Ruska and Max Knoll coupled two electron lenses, creating an initial microscope with significantly superior performance to that of optical microscopes. In 1933, Ruska joined Siemens, leading the development of the first commercially produced electron microscope in 1939 [24]. In the same year, Helmut Ruska successfully observed key features of TMV, such as its rod-like shape, length, outer diameter, and inner cavity, using electron microscopy. This breakthrough provided a powerful tool for the morphological study of viruses and cellular structures [25,26]. In 1937, Frederick Bawden and Norman Pirie further revealed the structure of TMV particles, which were approximately 95% protein and 5% RNA. These particles form rods capable of yielding liquid crystals with a regular substructure detectable via X-ray diffraction [27]. This finding indicated that viruses are molecules with biological characteristics and thus contributed to the understanding of viral replication mechanisms and infectivity.

In 1937, Max Delbruck and Emory Ellis clarified the dynamics of the phage reproductive cycle, including adsorption, latency, synthesis, and cleavage, revealing the interactions and replication processes between phages and bacteria. These researchers demonstrated the “one-step growth curve” (single-burst experiment), which made it possible to study phage reproduction in a single cell and provided the basis for subsequent research in the fields of genetics and molecular biology, especially on the importance of DNA and the mechanism of genetic information transfer; later, phages were often used as model organisms for genetic research [28]. In 1946, Joshua Lederberg at Yale University and his research group discovered that certain strains of bacteria could reprogram their genetic material, contradicting the common assumption that bacteria were primitive organisms unsuitable for genetic analysis. These researchers demonstrated that bacteria could serve as a powerful experimental system, entered the field of virology, such as transduction using phages, and later developed their a method for their application as a tool for bacterial genetics [29].

In 1950, Andre Lwoff and colleagues unveiled the mechanism of lysogeny. Lysogeny refers to the state in which a bacteriophage, upon infecting a bacterium, integrates its genetic material into the host bacterial chromosome, establishing a stable coexistence. In this scenario, the bacterium can continue to replicate while harboring the viral genes without causing lysis of the host cell. This is distinct from lytic infection, in which bacterial lysis occurs, releasing new viral particles. Through ultraviolet illumination-induced production of lysogens in *Bacillus gigantilus*, the researchers revealed the perpetuation of the phage genome in bacteria during the lysogenic state, prompting further exploration of microscopic inheritance mechanisms and the genetic basis of phage induction [30].

In 1952, Alfred Hershey and Martha Chase used the T2 phage to design the famous “phage infection of bacteria experiment”, which involved the use of radioactive tracers to label phages and trace the transfer of proteins and DNA between the virus and its host, and thus demonstrated that DNA was the carrier of genetic information. This discovery was a powerful challenge to the prevailing protein hypothesis at the time and provided solid evidence to reveal the key properties of DNA as genetic material, which had a profound impact on subsequent genetic and molecular biology research. In the same year, Renato Dulbecco selected Western equine encephalitis virus as the object of study and applied phage plaque technology to animal virology, making quantitative detection and research of animal viruses possible [31].

The development of the inactivated poliomyelitis vaccine (IPV) by Jonas Salk in 1954 and the development of an oral polio vaccine (OPV) by Albert Sabin’s in the late 1960s were pivotal. The IPV, grown in monkey kidney cells, showed high efficacy, leading to an 80–90% reduction in the polio incidence within two years, and the OPV developed by Sabin, which is cost-effective, became a cornerstone in global polio prevention efforts.

From Stanley’s crystallization of a virus to the application of electron microscopy techniques, the field of virology made key advances during this period. The structural analysis of TMV and the development of a yellow fever vaccine led to a deeper understanding of the biological characteristics and replication mechanisms of viruses. Pioneering biochemical research laid the foundation for the development of molecular biology and provided new technical means for disease control and virus prevention.

## 4. Genetics Period (1955–1984)

In 1955, Heinz Conrat and Robley Williams achieved a groundbreaking feat by reassembling the nucleic acid and protein subunits of TMV into infectious particles, revealing novel mechanisms for genetic information transfer [32]. In 1956, Alfred Schramm and Fraenkel Conral independently demonstrated that the infectivity of TMV can be attributed to RNA molecules, challenging the prevailing protein-centric hypothesis. Their work highlighted the crucial role of TMV RNA in the transmission of genetic information and the synthesis of new viral particles, challenging the existing paradigms and significantly impacting the understanding of viral processes at the time [33].

In 1957, Alick Isaacs and Jean Lindenmann made a landmark discovery, unveiling viral interference by demonstrating that prior exposure to heat-inactivated viruses could confer resistance to subsequent infections. This discovery introduced the concept of interferon, a pivotal milestone in the biological sciences [34].

In the mid-1950s, the link between viruses and cancer gained prominence. In 1957, under Renato Dulbecco’s guidance, Howard Temin discovered that Rous sarcoma virus (RSV) could transform cells through quantitative cell culture in sparse layers, marking the inception of modern tumor virology [35].

The 1960s witnessed significant strides. In 1962, Daniel Nathans conducted successful in vitro translation experiments of phage RNA, revealing the excitatory effect of RNA. By 1967, Goulian and Koenberg replicated the DNA of phage ΦX174 in vitro, laying the groundwork for synthetic biology [36]. In 1968, Peter Duesberg’s research on the multimolecular nature of influenza virus RNA inspired subsequent investigations into the genetic mechanisms and evolution of influenza viruses.

In 1970, David Baltimore and Howard Temin independently discovered reverse transcriptase, revealing how RNA viruses replicate genetic material through DNA. This breakthrough complemented the central dogma of molecular biology. The findings showed that DNA provided the information that was transcribed into mRNA, which was then used for translation into proteins, providing insights into viral replication mechanisms and diseases. Baltimore’s discovery of RNA polymerase and classification of viral genetic systems based on genome types expanded the understanding of this process [37]. Based on the replication strategy of viruses and the process of RNA–DNA transcription, viruses were categorized into seven groups, which encompass different types, such as positive-sense RNA, negative-sense RNA, double-stranded RNA, and reverse transcription processes. This classification system provides a crucial framework for the systematic study of virology. The 1970s witnessed pioneering strides in genetic engineering. In 1972, Paul Berg’s exploration of gene regulatory mechanisms in mammalian cells led to the creation of the first recombinant DNA molecule by splicing *E. coli* plasmids with SV40 viral DNA. In 1973, Nathans used restriction endonucleases to cut SV40 DNA into specific segments, establishing the first viral genome map [38,39].

In 1977, Fred Sanger and his team revolutionized DNA sequencing with the dideoxy strand termination method, earning him the 1980 Nobel Prize in Chemistry [40,41]. Concurrently, Phillip Sharp and Richard Roberts discovered RNA splicing, challenging the conventional notions of genetic information continuity. Their study of adenovirus gene transcription revealed that adenovirus mRNA corresponded to discontinuous DNA segments, transforming the understanding at the time and paving the way for split gene discoveries [42].

In 1978, Stephen Harrison and colleagues determined the atomic structure of tomato yellow dwarf virus, establishing the structure of icosahedral plant viruses. In 1985, scientists elucidated the structure of poliovirus with a resolution of 2.9 Å. The availability of vaccines enabled researchers to crystallize a more stable viral strain for experimental purposes. This achievement not only advanced studies on virus assembly, evolution, immunology, and antiviral drug design but also contributed to a better understanding of the pathogenic mechanisms of poliovirus. However, scientists at that time faced technical bottlenecks in high-throughput protein crystal screening and were thus continuously seeking more efficient and broad-spectrum protein crystallization methods to propel advancements in the field of structural biology [43].

Stanley Prusiner’s prion theory explains transmissible spongiform encephalopathy, revolutionizing the study of protein aggregation in conditions such as Alzheimer’s disease and Parkinson’s disease [44]. Prions are composed of misfolded proteins, lack nucleic acids, are incapable of self-replication, and can propagate diseases by inducing the misfolding of normal proteins. Viroids, another type of pathogenic particles, consist of short, single-stranded RNA molecules without a protein coat. The biological characteristics and definitions of prions and viroids became further understood and elucidated by virology and life science studies.

In 1983, Francoise Sinoussi, Luc Montagnier, and Robert Gallo independently discovered human immunodeficiency virus (HIV-1), laying the foundation for AIDS research. This breakthrough led to the development of diagnostic tests, prevention strategies, control policies, and antiviral drugs [45]. Concurrently, Zur Hausen and Gissmann isolated HPV subtypes 16 and 18 from genital warts in the early 1980s, revealing their link to approximately 75% of cervical cancers. This pivotal discovery sparked debates on the origins of cervical cancer and paved the way for subsequent HPV vaccine development [46].

In the genetics phase, pivotal advances highlighted the role of genetic mechanisms in virology. The core principles of molecular biology emphasize the central role of DNA in genetic information. Breakthroughs, such as in vitro translation experiments and the discovery of reverse transcriptase, have provided vital insights into virus life cycles and replication. The identification of RNA splicing transformed the understanding of gene expression, and DNA sequencing methods, such as the Sanger method, have catalyzed the development of genomics. This era laid a robust foundation, impacting biology and medicine and paving the way for molecular advancements in virology.

## 5. Molecular Biology Period (1985-)

In 1985, George Smith introduced phage display technology, which merged foreign protein-coding genes with phage structural genes. This innovation enabled the presentation of fusion proteins on the phage surface, revolutionizing antibody engineering, antigen determination, vaccine development, and drug screening applications [47].

Additionally, Maurice Hilleman achieved a milestone in 1986 with the FDA approval of the genetically engineered hepatitis B vaccine. By inserting the hepatitis B surface antigen (HBsAg) gene into yeast cells, a noninfectious surface protein was produced, constituting a groundbreaking achievement in genetic engineering for vaccine production [48].

In the late 1980s, Manfred Eigen and John Holland coined the term “pseudopopulation” to explain closely related viral variants within RNA virus populations due to their high mutation rate. This genetic diversity underscores their adaptability to environmental changes, leading to drug resistance and vaccine evasion. The dynamic distribution of variants shapes the evolutionary trajectory of RNA viruses, making them formidable adversaries in the face of immune responses and therapeutic interventions [49].

In 1990, French Anderson initiated the first legal retroviral-based gene therapy trial for ADA deficiency, marking the onset of gene therapy research. Simultaneously, Zylafudine (AZT) synthesized by Jerome Horwitz in 1964 underwent clinical trials in the mid-1980s, which demonstrated its safety in HIV patients and its ability to enhance the CD4+ T-cell counts [50]. In the late 1980s, Martin Hirsch performed foundational and basic research into combination antiviral treatment for HIV infection, which evolved into highly active antiretroviral therapy (HAART) proposed by David Ho in 1996. The introduction of HAART reduced the AIDS death rates by 50%, ushering in a transformative era in HIV treatment [51].

In 1994, Karl-Klaus Conzelmann performed rabies virus reverse genetics using plasmids for antigenome RNA transcription per gene, not genome RNA. This breakthrough enabled precise RNA virus genome manipulation, providing a vital platform for in-depth genomics research, viral mutant creation for pathogenesis studies, and the use of these viruses as vectors for foreign protein expression. Conzelmann’s approach significantly advanced RNA virus research applications [52].

In 2002, Eckard Wimmer’s team synthesized poliovirus DNA, a milestone in synthetic biology that triggered ethical debates on the biosynthesis of pathogens. Concurrently, Joseph DeRisi and Ian Lipkin developed the Virochip and GreeneChip microarray platforms in 2002, revolutionizing pathogen identification and enhancing epidemic prevention and virology research [53,54]. In 2003, the Lawrence Livermore National Laboratory created the autonomous pathogen detection system (APDS), which was designed to offer early warnings of biological threats via aerosol transmission. The core technology of APDS, which was based on the Luminex assay platform and flow cytometry, can simultaneously detect multiple pathogen markers, which represents a significant advancement in automated virus detection for biosecurity and public health.

In 2005, Charles Rice replicated HCV in cell culture, discovering drug targets and pioneering the antiviral drug sofosbuvir, which was approved in 2013. The success of sofosbuvir has increased the cure rate for hepatitis C patients to above 95%, significantly alleviating the global HCV burden [55].

In the late 20th century, CRISPR/Cas9 genome editing, which was originally discovered in bacteria and archaea, became a potent gene-editing tool through the work conducted by Yoshizumi Ishino, Feng Zhang, Emmanuelle Charpentier, and Jennifer DoudnaYoshizumi in 2013. CRISPR/Cas9, which is highly specific and flexible, has proven effective for identifying and cutting viral genomes and thus offers a potential solution to combat viral infections [56].

In 2014, ZMapp, a genetically engineered monoclonal antibody drug, was developed, and this drug exhibited efficacy in treating Ebola virus infection in primates, demonstrating the potential of genetic engineering for antiviral drug development. Nobel laureates Eric Betzig, Stefan Hell, and William Moerner received recognition in 2014 for their work on super-resolved fluorescence microscopy [57]. This breakthrough empowered scientists to observe cellular structures and processes at an unprecedented level, enabling the study of cellular components at resolutions below 10–100 nanometers, and opened new avenues for understanding complex molecular mechanisms, protein–protein interactions, and dynamic processes within living cells. In 2016, Mary Estes introduced enteroids, a technique for growing challenging viruses such as human norovirus. “Enteroids” are three-dimensional tissue structures derived from isolated stem cells or stem cell derivatives obtained from intestinal tissues. This cell culture technique mimics the intricate structure and physiological environment of the human intestinal tract. This innovation provided a crucial platform for the preclinical evaluation of vaccines, antivirals, and other treatments and thus offered new strategies against viral infections [58].

The pinnacle of innovation occurred in 2020 with the development of mRNA vaccine technology, and this technology allowed the major breakthrough of producing a COVID-19 vaccine in under eight months. This remarkable feat set a record and revolutionized vaccine development, sparking interest in the application of RNA vaccines to other diseases. The advantages of this technology, including rapid production, a targeted immune response, and reduced side effects, make it a significant innovation in the vaccine field [1,2]. In 2020, a collaborative effort between teams led by Sai Li from Tsinghua University and Lanjuan Li from Zhejiang University involved the use of cryo-electron tomography and subtomogram averaging techniques. This groundbreaking study provided the first comprehensive discovery of the authentic three-dimensional structure of COVID-19 from the inside out. This research provided in situ insights into the native conformation and distribution of the spike proteins on the virus surface. Furthermore, mass spectrometry analysis elucidated the in situ glycosylation composition of viral spike proteins. These findings have significant implications for the development of inactivated virus vaccines, in vitro recombinant vaccines, and antibody therapies [59].

During the molecular biology period, key advancements, such as phage display technology, genetic engineering of the hepatitis B vaccine, the viral resistance of CRISPR/Cas9, and the use of retroviruses as vectors, have led to the profound integration of virology with molecular biology. The swift progress in COVID-19 vaccine development and the establishment of autonomous pathogen detection systems offer crucial tools to combat infectious diseases. These breakthroughs, which foster interdisciplinary collaboration, novel vaccine strategies, and effective treatments, have garnered global attention and provide robust support for the ongoing evolution of virology in addressing infectious disease challenges.

## 6. Summary

**Figure 1:**
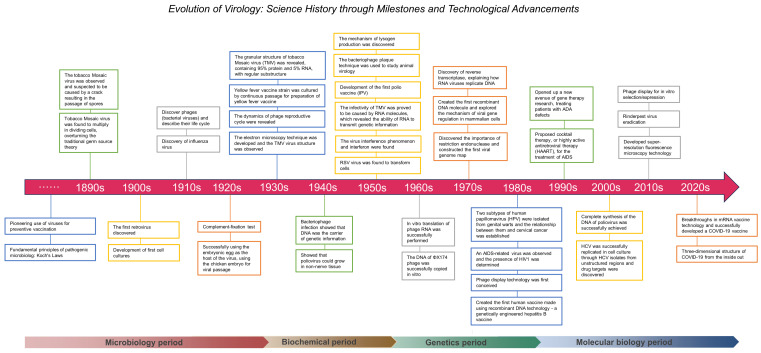
History of virology from the late 19th century to the 2020s, which encompasses four periods, namely, the microbiology, biochemistry, genetic, and molecular biology periods. The timeline provided in the figure briefly describes the important achievements and technological advancements in each period.

The evolution of virology unfolded across four periods (Figure 1). In the microbiology phase, early vaccine endeavors addressed infectious diseases, but a complete understanding of viruses was hindered by limited microscopy resolution, which prevented detailed observations of their structure and behavior. Uncertainty regarding the composition and nature of viruses prevailed, sparking debates about their living status. Phage studies provided critical insights into viral replication and heritability. During the transition to the biochemical period, techniques were developed, and the ability to understand the molecular mechanisms and biochemical characteristics of viruses became a major breakthrough. Breakthroughs in electron microscopy enhanced the comprehension of viral traits, while revelations that viruses are composed of proteins or protein-related substances sparked profound discussions on the nature of life. Vaccines for polio and yellow fever marked advancements during this phase. The genetics phase underscored the centrality of genetic information transmission. Scientists unveiled the roles of viral RNA and DNA, fostering the rise of molecular biology. Discoveries during this phase, such as HIV-1 and HPV, significantly impacted medicine and virology, advancing the understanding of virus-related diseases and vaccine development. In the molecular biology phase, genetic and protein engineering have burgeoned, enabling innovations in gene editing, vaccine preparation, and antiviral strategies. Retroviruses have facilitated gene delivery, leading to the emergence of genetic modification and gene therapy. Advances such as CRISPR/Cas9, cocktail therapy, prion theory, and microarray technology have revolutionized treatment and virus detection, marking the culmination of virological progress.

High-throughput sequencing transforms viral genomics by employing techniques such as whole-genome sequencing and meta-transcriptomics to obtain profound insights into the structure, evolution, and function of viruses. Evolutionary studies decipher the adaptive mechanisms of viruses, predicting their public health impacts. Synthetic biology constructs functional viral particles, advancing the development of vaccines and antiviral drugs. Ethical considerations in virology research call for a balance between technological innovation and safety standards to ensure public well-being. Current research not only deepens the scientific understanding but also prompts reflections on the ethics and safety of scientific endeavors.

The exploration of the history of virology reveals a dynamic evolution through four transformative periods that were marked by groundbreaking breakthroughs in microbiology, biochemistry, genetics, and molecular biology. These advancements not only reshape the scientific landscape but also profoundly impact medicine, public health, and life sciences. The interdisciplinary synergy underlying each period establishes a robust foundation for the growth of the virology field, offering valuable resources for future disease challenges. The historical journey from early virus discovery to intricate studies of the structure and genetic mechanisms of viruses inspires further exploration of the uncharted territories in the field of virology. International cooperation and collaborative efforts play a pivotal role in facilitating major discoveries through data sharing and technology exchange. The constant innovation of technological tools, such as electron microscopy and high-throughput sequencing, has enriched the research on virology. Embracing emerging technologies ensures sustained advancements, contributing to the ongoing construction of virology and enhancing research capabilities.

Overall, the development of virology has injected new vitality into societal health, technological innovation, and international collaboration, making substantial contributions to global sustainable development. First, virology has provided profound theoretical foundations and practical guidance for the prevention and control of infectious diseases. In-depth studies of the structures, life cycles, and transmission pathways of different viruses have enabled scientists to formulate more effective strategies. The widespread use of vaccines globally has significantly reduced the incidence and mortality rates of infectious diseases. Second, advancements in virology have propelled rapid developments in the fields of biotechnology and genetic engineering. A profound understanding of the genetic structures and replication mechanisms of viruses has provided crucial knowledge on areas such as gene editing and gene therapy. By leveraging viral vector systems, scientists can precisely manipulate genes, leading to innovations in various fields such as medical treatments and agricultural improvements. Third, the development of virology underscores the importance of close international collaboration in the prevention and research of infectious diseases. Faced with global pandemic challenges, scientists, medical experts, and government agencies from various countries collaborate, sharing information, experiences, and research outcomes. This international cooperation not only enhances the monitoring and control of diseases but also accelerates the development of vaccines and treatment methods. Particularly during the COVID-19 pandemic, virology has become a global focal point, emphasizing that international collaboration is crucial for effectively combating pandemics.

## Data Availability

Not applicable.
